# Conventional versus advanced imaging selection for endovascular treatment of basilar artery occlusion strokes

**DOI:** 10.1093/esj/23969873251364973

**Published:** 2026-01-01

**Authors:** Huanwen Chen, Marco Colasurdo, Hidetoshi Matsukawa, Conor Cunningham, Ilko Maier, Sami Al Kasab, Pascal Jabbour, Joon-Tae Kim, Stacey Quintero Wolfe, Ansaar Rai, Robert M Starke, Marios-Nikos Psychogios, Edgar A Samaniego, Nitin Goyal, Shinichi Yoshimura, Hugo Cuellar, Jonathan A Grossberg, Ali Alawieh, Ali Alaraj, Mohamad Ezzeldin, Daniele G Romano, Omar Tanweer, Justin Mascitelli, Isabel Fragata, Adam Polifka, Fazeel Siddiqui, Joshua Osbun, Roberto Crosa, Charles Matouk, Min S Park, Michael R Levitt, Waleed Brinjikji, Mark Moss, Travis Dumont, Ergun Daglioglu, Richard Williamson, Pedro Navia, Reade De Leacy, Shakeel Chowdhry, David J Altschul, Alejandro M Spiotta, Peter Kan

**Affiliations:** National Institute of Neurological Disorders and Stroke, National Institutes of Health, Bethesda, MD, USA; MedStar Georgetown University Hospital, Washington, DC, USA; Oregon Health & Science University Hospital, Portland, OR, USA; Medical University of South Carolina, Charleston, SC, USA; Medical University of South Carolina, Charleston, SC, USA; Universitätsmedizin Göttingen, Göttingen, Germany; Medical University of South Carolina, Charleston, SC, USA; Thomas Jefferson University, Philadelphia, PA, USA; Chonnam National University Hospital, Gwangju, Republic of Korea; Wake Forest Baptist Health, Lexington, NC, USA; West Virginia University, Morgantown, WV, USA; University of Miami Health System, Miami, FL, USA; Universitätsspital Basel, Basel, Switzerland; University of Iowa, Iowa City, IA, USA; University of Tennessee Health Science Center, Semmes Murphey Foundation, Memphis, TN, USA; Hyogo College of Medicine, Hyogo, Japan; LSU Health Shreveport, Shreveport, LA, USA; Emory University, Atlanta, GA, USA; Emory University, Atlanta, GA, USA; University of Chicago at Illinois, Chicago, IL, USA; University of Houston, HCA Houston Healthcare Kingwood, Kingwood, TX, USA; Aou S. Giovanni di Dio e Ruggi d’Aragona, Salerno, SA, Italy; Baylor College of Medicine, Houston, TX, USA; University of Texas Health Science Center at San Antonio, San Antonia, TX, USA; NOVA Medical School, Universidade Nova de Lisboa, Lisboa, Portugal; University of Florida, Gainesville, FL, USA; University of Michigan Health West, Wyoming, MI, USA; Washington University in St. Louis, St. Louis, MO, USA; Médica Uruguaya, Montevideo, Uruguay; Yale University, New Haven, CT, USA; University of Virginia, Charlottesville, VA, USA; University of Washington, Seattle, WA, USA; Mayo Clinic in Minnesota, Rochester, MN, USA; Washington Regional Medical Center, Fayetteville, AR, USA; University of Arizona, Tucson, AZ, USA; Health Science University, Ankara Bilkent City Hospital, Ankara, Turkey; Alleghany Hospital, Pittsburgh, PA, USA; Hospital Universitario La Paz, Madrid, Spain; Mount Sinai Health System, New York, NY, USA; North Shore University Health System, Evanston, IL, USA; Montefiore Medical Center, Albert Einstein College of Medicine, Bronx, NY, USA; Medical University of South Carolina, Charleston, SC, USA; University of Texas Medical Branch at Galveston, Galveston, TX, USA

**Keywords:** Basilar, stroke, thrombectomy, imaging, magnetic resonance, perfusion, hemorrhage, time window, computed tomography, ASPECT

## Abstract

**Introduction:**

Endovascular thrombectomy (EVT) is an effective treatment for basilar artery occlusion (BAO) stroke in select patients. While there is a growing body of literature suggesting that advanced imaging modalities such as computed tomography perfusion (CTP) and magnetic resonance (MR) may not be necessary for selecting anterior circulation large vessel occlusion stroke patients for EVT, whether advanced imaging may be superior to conventional imaging (non-contrast CT and CT angiography) in identifying good treatment candidates among BAO patients is less clear.

**Patients and methods:**

This was a multicenter retrospective cohort study of BAO EVT patients treated from 2013 to 2022 in the Stroke Thrombectomy and Aneurysm Registry. Patients selected for EVT by advanced imaging (CTP or MR) were matched with those selected by conventional imaging using propensity score matching (PSM) accounting for possible confounders. Primary outcome was functional independence at 90 days. Other outcomes include bedridden state or death at 90-days and symptomatic intracranial hemorrhage (sICH).

**Results:**

268 patients were included. 150 patients were selected for BAO EVT by conventional imaging, 86 by CTP, and 32 by MR. Patients selected by advanced imaging were significantly older than those selected by conventional imaging (median age 71 vs 64 years, *p* = 0.001); patient characteristics were otherwise similar between cohorts. After PSM, 90-day outcomes were similar between the two cohorts (*p* = 0.56), with similar rates of functional independence (39.4% vs 35.1%, *p* = 0.65), bedridden state or death (40.4% vs 44.7%, *p* = 0.66), and sICH (3.3% vs 5.7%, *p* = 0.49) for conventional and advanced imaging groups, respectively. Results were similar across treatment time windows (all *p* > 0.05).

**Conclusions:**

Selecting patients for basilar EVT using conventional versus advanced imaging did not result in different clinical outcomes, regardless of treatment time windows. Conventional imaging appears sufficient as a first-line tool for selecting basilar EVT patients in routine clinical practice.

## Graphical Abstract

**Graphical abstract:**
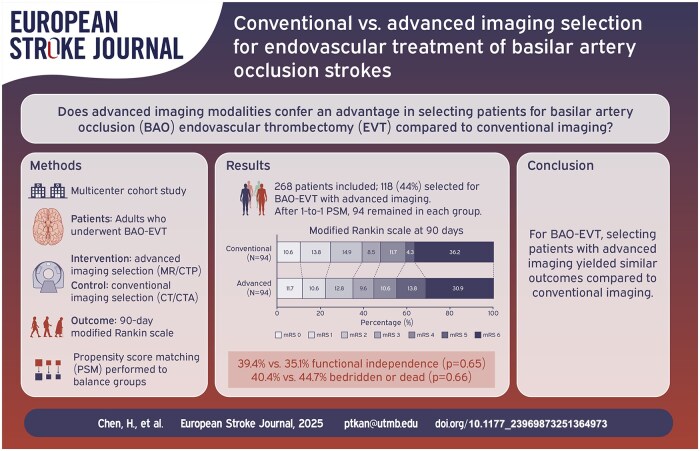

## Introduction

Four landmark randomized controlled trials have investigated the efficacy and safety of endovascular thrombectomy (EVT) for acute ischemic stroke due to basilar artery occlusion (BAO).^1–4^ While pooled analyses demonstrated overall treatment benefit of BAO-EVT, only two of the four trials (ATTENTION and BAOCHE) met their primary efficacy endpoint.^5^ The heterogeneity of basilar EVT’s treatment benefit may be due in part to differences in radiographic features of trial participants.^6^ In the ATTENTION and BAOCHE trials, a minority of patients were selected for trial inclusion based on magnetic resonance (MR)^2,3^; however, whether these patients had different outcomes compared patients selected by conventional computed tomography (CT) and CT angiography (CTA) was not reported. To date, whether advanced imaging modalities such as MR or computed tomographic perfusion (CTP) confer an advantage over conventional CT/CTA when selecting BAO-EVT patients is overall unclear.

In this retrospective analysis of an international, multi-center database of endovascular stroke treatments, we investigate clinical outcomes of BAO-EVT patients selected by conventional imaging versus advanced imaging modalities. Given that advanced imaging modalities may not confer a significant clinical advantage in selecting anterior circulation EVT patients,^7–12^ we hypothesize that patient selection using conventional neuroimaging may yield similar clinical outcomes compared to advanced modalities for BAO-EVT patients.

## Methods

### Database characteristics

This was a retrospective cohort study of the Stroke Thrombectomy and Aneurysm Registry (STAR).^13^ The registry includes centers from the U.S, Europe, South America, and Asia. A database of stroke patients who underwent EVT at 32 stroke centers participating in STAR from January 2013 to December 2022 was used for the current study. The study was approved by the institutional review board at each participating institution; patient consent was waived. The data at each institution were obtained retrospectively and collected according to a standardized protocol. Verification, de-identification, and attestation of data accuracy were performed by investigators at each contributing institution. Individual patient data from each contributing institution were pooled by investigators at STAR.

### Patients and clinical variables

Adult patients who underwent EVT for BAO with available information on imaging modality used for patient selection were included. Exclusion criteria include: (1) concomitant anterior circulation vascular occlusion, (2) lack of information regarding imaging modality used for treatment selection, (3) treatment selection based on imaging modality other than CT/CTA, CTP, or MR, and (4) lack of 90-day clinical follow-up. Patients were divided into two study cohorts: Those selected for treatment by conventional imaging (CT and CTA) and those selected by advanced imaging (CTP or MR). The choice of imaging modality and processing software for patient selection was per local institutional protocols. The reasons underlying the choice of front-line imaging modality and criteria for pursuing additional second-line imaging are not recorded in the STAR database.

Patient demographic data included age, sex, medical comorbidities, and pre-stroke disability measured by modified Rankin scale^14^ (mRS). Clinical characteristics included admission National Institutes of Health stroke scale (NIHSS), administration of intravenous thrombolysis, additional sites of vascular occlusion (posterior cerebral artery or vertebral artery), and symptom onset or last-known-well time to arteriotomy were also captured. Procedural data included additional endovascular procedures (angioplasty, intracranial stenting, or intra-arterial thrombolysis) and successful revascularization (modified treatment in cerebral ischemia score^15^ of 2b or greater).

### Study outcomes

Primary study outcome was the rate of good 90-day (±20 days) outcomes (mRS 0-2, implying functional independence). Secondary outcomes included rates of acceptable 90-day outcomes (mRS 0-3, implying ambulatory independence), poor 90-day outcomes (mRS 5 or 6, implying bedridden state or death), any intracranial hemorrhage (ICH), and symptomatic ICH (sICH, defined as presence of ICH and neurological worsening of 4 points or greater on the NIHSS).^16^

### Statistical analysis

Descriptive statistics were presented as median (Q1-Q3) for continuous variables or percentage for categorical variables. Missing data for pre-stroke mRS were imputed with zero (assuming no pre-existing disability), and missing data for prior intravenous thrombolysis were imputed as no (assuming no prior treatment).

Propensity score matching (PSM) was performed to balance the conventional and advanced imaging cohorts. Propensity scores were calculated using a binary logistic regression model including all captured clinical variables including sex, age, medical comorbidities, pre-stroke disability, admissions NIHSS, additional sites of vascular occlusion, intravenous thrombolysis treatment, time from stroke onset to arteriotomy (categorized into: 0–6 h, 6–12 h, 12–18 h, 18–24 h, more than 24 h, or unknown), additional endovascular procedures, and successful revascularization. Then, patients from the advanced imaging cohort were matched with patients from the conventional imaging cohort based on propensity scores with one-to-one nearest neighbor matching and a maximum allowable distance of 0.1. PSM performance was evaluated by standardized mean differences (SMD) of matched variables, where SMD less than 0.1 was deemed adequately balanced.

Primary and secondary outcomes were compared between the PSM cohorts with Fisher’s exact tests. Ordinal regression analysis was used to compare 90-day mRS outcomes between groups, and tests of parallel lines were used to confirm that the proportional odds assumption was not violated. For doubly robust analyses, additional adjustments of confounders were made for variables that remain unbalanced after PSM (SMD 0.1 or greater) as well as known predictors for post-EVT outcomes (age, admission NIHSS, pre-stroke mRS, and time from stroke onset to arteriotomy)^17^ using logistic regression models assessing differences in clinical outcomes between the PSM cohorts. Subgroup analyses include patients treated in the early window (time from stroke onset to arteriotomy within 6 h), patients treated in the late window (time from stroke onset to arteriotomy beyond 6 h or unknown), patients selected by CTP (and their corresponding PSM patients who underwent conventional imaging), and patients selected by MR (and their corresponding PSM controls).

*p*-Value less than 0.05 was deemed statistically significant for the primary outcome. Adjustments for multiple comparisons were not performed for secondary outcomes, subgroup analyses, or sensitivity analyses due to the explorative nature of these additional comparisons. Statistical analyses were conducted using SPSS v29.0.

## Results

A total of 373 adult patients were identified for inclusion. Nine patients with concomitant anterior circulation vascular occlusion, 48 without information on post-EVT revascularization, 41 without information on 90-day outcomes, and 7 selected for treatment with an imaging modality other than CT/CTA, CTP or MR were excluded. A total of 268 were included in the study, of whom 150 were selected for basilar EVT using conventional imaging, and 118 using advanced imaging (86 by CTP, and 32 by MR). The study flowchart is presented in Supplemental Figure S1.

### Patient characteristics

Patient demographic, clinical and procedural characteristics are shown in Table 1. Patients selected for treatment by conventional imaging were significantly younger than those selected by advanced imaging (median age 64 vs 71 years, *p* < 0.001), and less likely to have been treated with angioplasty (2.7% v. 9.3%, *p* = 0.029). Patient characteristics were otherwise not significantly different between the two groups.

**Table 1.** Patient characteristics.

Characteristics – Median (Q1–Q3) or % (*n*)	All patients (*n* = 268)	Unmatched cohorts			PSM cohorts			
		Conventional imaging (*n* = 150)	Advanced imaging (*n* = 118)	*p*-Value	Conventional imaging (*n* = 94)	Advanced imaging (*n* = 94)	*p*-Value	SMD
Male sex	63.1 (169)	64.0 (96)	61.9 (73)	0.80	60.6 (57)	62.8 (59)	0.88	0.044
Age (years)	68 (56–78)	64 (53–75)	71 (60–80)	0.001	68 (59–78)	70 (60–80)	0.56	0.113
Type 2 diabetes mellitus	31.7 (85)	29.3 (44)	34.7 (41)	0.36	28.7 (27)	35.1 (33)	0.43	0.137
Hypertension	73.9 (198)	74.0 (111)	73.7 (87)	1.00	75.5 (71)	75.5 (71)	1.00	<0.001
Atrial fibrillation	27.6 (74)	25.3 (38)	30.5 (36)	0.41	29.8 (28)	34.0 (32)	0.64	0.091
Hyperlipidemia	50.4 (135)	48.7 (73)	52.5 (62)	0.54	47.9 (45)	53.2 (50)	0.56	0.107
Heart failure	16.4 (43)	17.4 (25)	15.3 (18)	0.74	17.0 (16)	16.0 (15)	1.00	0.029
Pre-stroke mRS^a^	0 (0–1)	0 (0–1)	0 (0–1)	0.77	0 (0–1)	0 (0–1)	0.30	0.063
Admission NIH Stroke Scale	18 (8–27)	19 (9–27)	17 (8–27)	0.61	17 (9–26)	17 (8–26)	0.88	0.068
Imaging modality								
CT/CTA	56.0 (150)	100.0 (150)	-	-	100.0 (94)	-	-	-
CT perfusion	32.1 (86)	-	72.9 (86)		-	75.5 (71)		
MR	11.9 (32)	-	27.1 (32)		-	24.5 (23)		
Additional sites of vascular occlusion								
PCA	8.2 (22)	10.0 (15)	5.9 (7)	0.27	5.3 (5)	7.4 (7)	0.77	0.087
Vertebral	4.1 (11)	3.3 (5)	5.1 (6)	0.54	5.3 (5)	5.3 (5)	1.00	<0.001
Prior IVT^b^	23.1 (62)	21.3 (32)	25.4 (30)	0.47	19.1 (18)	21.3 (20)	0.86	0.053
Stroke onset to arteriotomy time								
0–6 h	44.0 (118)	46.7 (70)	40.7 (48)	0.45	43.6 (41)	43.6 (41)	0.99	0.109
6–12 h	18.3 (49)	18.0 (27)	18.6 (22)		18.1 (17)	17.0 (16)		
12–18 h	15.7 (42)	16.7 (25)	14.4 (17)		19.1 (18)	18.1 (17)		
18–24 h	2.6 (7)	3.3 (5)	1.7 (2)		3.2 (3)	2.1 (2)		
More than 24 h	4.1 (11)	2.7 (4)	5.9 (7)		3.2 (3)	4.3 (4)		
Unknown	15.3 (41)	12.7 (19)	18.6 (22)		12.8 (12)	14.9 (14)		
Additional endovascular procedures								
Angioplasty	5.6 (15)	2.7 (4)	9.3 (11)	0.029	3.2 (3)	3.2 (3)	1.00	<0.001
Intracranial stenting	6.0 (16)	4.7 (7)	7.6 (9)	0.44	3.2 (3)	4.3 (4)	1.00	0.056
IA-tPA	6.3 (17)	4.7 (7)	8.5 (10)	0.22	6.4 (6)	7.4 (7)	1.00	0.042
Successful recanalization	89.6 (240)	91.3 (137)	87.3 (103)	0.32	91.5 (86)	90.4 (85)	1.00	0.037

mRS: modified Rankin scale; CT: computed tomography; CTP: CT perfusion; MR: magnetic resonance; PCA: posterior cerebral artery; IVT: intravenous thrombolysis; PSM: propensity score-matched; IA-tPA: intra-arterial tissue-like plasminogen activator.

^a^Eleven patients had missing pre-stroke mRS information, and were imputed as zero.

^b^Four patients had missing prior-IVT information, and were imputed as no.

Patients in the two study arms were matched using propensity scores calculated with all captured clinical variables. After PSM, *n* = 94 patients remained in each arm, and there were no significant differences in patient characteristics. SMD measurements were acceptably low for all clinical variables except for age (SMD 0.113), type 2 diabetes mellitus (T2DM; SMD 0.137), hyperlipidemia (SMD 0.107), and time from stroke onset to arteriotomy (SMD 0.109).

### Patient outcomes

**Figure 1.**
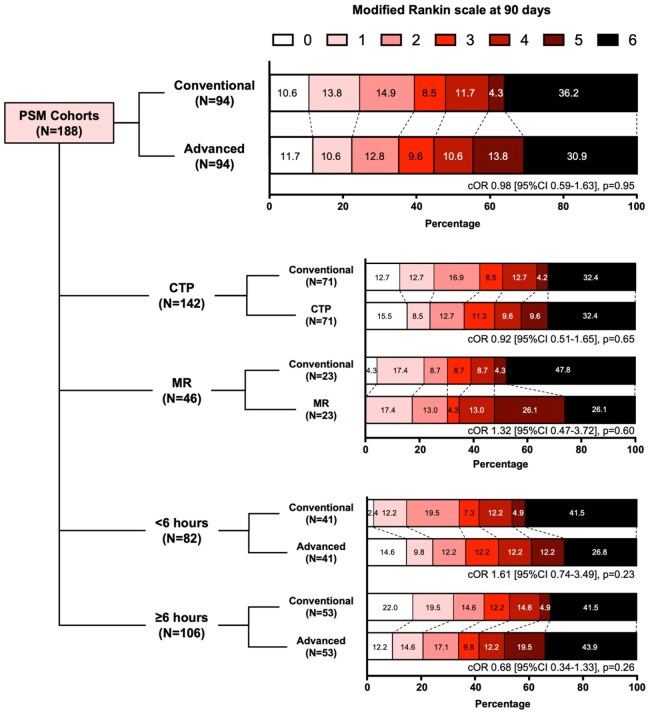
Ninety-day functional outcomes of basilar thrombectomy patients selected by conventional and advanced imaging in the propensity score-matched (PSM) cohorts and subgroups stratified by advanced imaging modality and time from stroke onset to arteriotomy. Patients who received computed tomography perfusion (CTP) or magnetic resonance (MR) imaging were compared with their PSM counterparts in the conventional imaging arm. Patients with unknown time of stroke onset were included in the ⩾6 h subgroup. Ordinal regression analyses were conducted for comparisons between groups; tests of parallel lines confirmed that proportional odds assumption was not violated. *p*-Values < 0.05 were deemed statistically significant.

Patient outcomes are shown in Table 2. Between the PSM cohorts, there were no statistically significant differences in terms of 90-day functional independence (39.4% vs 35.1%, *p* = 0.65), 90-day independent ambulation (47.9% vs 44.7%, *p* = 0.77), 90-day bedridden state or death (40.4% vs 44.7%, *p* = 0.66), ICH (9.7% vs 13.3%, *p* = 0.49), or sICH (3.3% vs 5.7%, *p* = 0.49; Table 2). Ninety-day outcomes measured by mRS were also not statistically different in ordinal regression analysis (common odds ratio [cOR] 0.98, 95% confidence interval [CI] 0.59 to 1.63, *p* = 0.95; Figure 1). After additional adjustments for age, NIHSS, pre-stroke mRS, time from stroke onset to arteriotomy, T2DM, and hyperlipidemia, advanced imaging selection was not associated with significantly different odds of functional independence (adjusted odds ratio (aOR) 0.80, [95% CI 0.40 to 1.60], *p* = 0.53), independent ambulation (aOR 0.84 [95% CI 0.43 to 1.63], *p* = 0.60), or bedridden state or death (aOR 1.22 [95% CI 0.64 to 2.32], *p* = 0.55).

**Table 2.** Patient outcomes of PSM cohorts without and with additional multivariable adjustments.

Outcome	Without additional adjustments			With additional adjustments	
	Conventional Imaging	Advanced Imaging	*p*-Value	OR for Advanced Imaging [95% CI]	*p*-Value
Functional independence	39.4 (37/94)	35.1 (33/94)	0.65	0.80 [0.40–1.60]	0.53
Independent ambulation	47.9 (45/94)	44.7 (42/94)	0.77	0.84 [0.43–1.63]	0.60
Bedridden state or death	40.4 (38/94)	44.7 (42/94)	0.66	1.22 [0.64–2.32]	0.55
Any ICH	9.7 (9/93)	13.3 (12/90)	0.49	-	
Symptomatic ICH	3.3 (3/91)	5.7 (5/88)	0.49	-	

ICH: intracranial hemorrhage; OR: odds ratio.

### Subgroup analyses

Subgroup analyses of CTP or MR selection are shown in Table 3. Patients selected by CTP or MR did not have significantly different 90-day clinical outcomes compared to their PSM counterparts. The imaging modality used for patient selection also did not lead to difference in patient outcomes for those treated in the early (within 6 h of stroke onset) or late (beyond 6 h or unknown time of stroke onset) time windows (Table 4). Distributions of 90-day mRS scores for subgroup analyses are presented in Figure 1, and ordinal regression analyses did not reveal any statistically significant differences in 90-day mRS across all comparisons (all *p* > 0.05).

**Table 3.** Subgroup analyses of CTP and MRI selected patients.

Outcome	CTP vs. PSM controls			MR vs. PSM controls		
	Conventional imaging	CT perfusion	*p*-Value	Conventional imaging	MR	*p*-Value
Functional independence	42.3 (30/71)	36.6 (26/71)	0.61	30.4 (7/23)	30.4 (7/23)	1.00
Independent ambulation	50.7 (36/71)	47.9 (34/71)	0.87	39.1 (9/23)	34.8 (8/23)	1.00
Bedridden state or mortality	36.6 (26/71)	42.3 (30/71)	0.61	52.2 (12/23)	52.2 (12/23)	1.00
Any ICH	10.0 (7/70)	7.5 (5/67)	0.77	8.7 (2/23)	30.4 (7/23)	0.14
Symptomatic ICH	4.3 (3/69)	3.0 (2/66)	1.00	0.0 (0/22)	13.6 (3/22)	0.23

CTP: computed tomography perfusion; MR: magnetic resonance; PSM: propensity score-matched; ICH: intracranial hemorrhage.

**Table 4.** Subgroup analyses of patients in the early (<6 h) and late (⩾6 h) time window.

Outcome	Early time window (<6 h)			Late time window (⩾6 h or unknown)		
	Conventional imaging	Advanced imaging	*p*-Value	Conventional imaging	Advanced imaging	*p*-Value
Functional independence	34.1 (14/41)	36.6 (15/41)	1.00	43.4 (23/53)	34.0 (18/53)	0.43
Independent ambulation	41.5 (17/41)	48.8 (20/41)	0.66	52.8 (28/53)	41.5 (22/53)	0.33
Bedridden state or mortality	46.3 (19/41)	39.0 16(41)	0.67	35.8 (19/53)	49.1 (26/53)	0.24
Any ICH	7.3 (3/41)	5.0 (2/40)	1.00	11.5 (6/52)	20.0 (10/50)	0.28
Symptomatic ICH	2.6 (1/39)	2.7 (1/37)	1.00	3.8 (2/52)	7.8 (4/51)	0.44

ICH: intracranial hemorrhage.

## Discussion

In this international multi-center retrospective study of BAO stroke patients, we found that conventional or advanced imaging selection of EVT patients did not result in significant differences in 90-day clinical outcomes or rates of ICH. These findings persisted in subgroup analyses across treatment time windows and advanced imaging modality used (CTP or MR). Overall, results of the current study suggest that conventional imaging (CT and CTA) may be sufficient for selecting BAO-EVT patients in routine clinical practice, and that the use of advanced imaging modalities such as CTP or MR may not be necessary.

The optimal use of advanced neuroimaging modalities such as CTP and MR during acute stroke triage to select patients for reperfusion therapy has been a topic of debate. In anterior circulation stroke, studies have demonstrated that EVT selection based on conventional CT alone may lead to similar outcomes, even in the extended time window.^7,12,18–21^ Recent positive results from low-ASPECTS thrombectomy trials for anterior circulation LVOs, negative trial results from distal and medium vessel occlusion strokes, and a growing body of literature advocating for direct-to-angiosuite systems may further obviate the need for advanced imaging during acute stroke triage in general.^18,21–25^ Despite these trends, whether advanced imaging selection for EVT for BAO strokes may be advantageous is less clear. CT-based imaging markers are important for selecting basilar EVT patients, and scoring systems are available for quantifying early ischemia in posterior circulation strokes using non-contrast CT (e.g. pc-ASPECTS^26^ and MPI^27^). However, unlike ASPECTS for anterior circulation strokes,^28^ pc-ASPECTS and MPI are lesser-known, and more prone to poor inter-rater reliability and signal artifacts in the posterior fossa.^29^ The heterogeneity of pc-ASPECTS areas in terms of clinical implications are also substantial (e.g. discrepancies in clinical deficit of pontine vs occipital lobe infarcts), further limiting its clinical use. Furthermore, while collaterals can be assessed for anterior circulation LVOs to assist patient selection, there is currently no validated tool to assess collateral status in the basilar territory. Thus, it is possible that advanced imaging modalities such as CTP and MR may provide more reliable tissue-level information, and their use may confer an advantage when optimizing patient selection.^30–33^

While some studies have suggested that CTP can identify hypo-perfused tissue for BAO strokes,^34–36^ the use of CTP in the posterior circulation is overall unvalidated and has not demonstrated clinical reliability in large clinical trials. In our study, CTP use did not appear to improve patient selection. Data on the reliability of CTP in the posterior circulation is limited,^32,37^ and the lack of significant difference in our study may be due to multiple factors. The reliability of CTP is known to be poor in subcortical areas (e.g. brain stem, thalami) compared to the cortex^38^; thus, CTP may not be as reliable for BAO strokes compared to anterior circulation occlusions. Beam hardening artifacts from the petrous bone may also limit the reliability of CTP in the brainstem.^29^ Furthermore, well-established thresholds for CTP metrics such as cerebral blood flow and time to peak were derived based on hemodynamic characteristics of the anterior cerebral circulation, and they may not be applicable to BAO strokes.^39,40^

Reasons underlying the lack of difference between conventional imaging and MR may be more nuanced. On one hand, DWI quantification of ischemic burden may not be reflective of permanent tissue damage, as these lesions can be reversible^41^ and may be more benign compared to ischemic changes on CT.^42^ Thus, MR-selected patients may be more likely to have favorable clinical outcomes. On the other hand, MR may have been selectively used as a second-line imaging modality at some institutions. Thus, some MR-selected patients may be associated with worse outcomes as they may have had limited or unfavorable CT scans. These possible and competing phenomena may have influenced the overall similar outcomes between patients selected for BAO-EVT by CT/CTA and MR in our study, and results must be interpreted with caution.

Despite the above uncertainties, our results suggest that BAO-EVT patients selected by CT/CTA in routine clinical practice have equivalent clinical outcomes compared to patients selected by CTP or MR. Given that it is less resource and time-intensive compared to advanced imaging modalities, CT/CTA may be appropriate and sufficient as a front-line selection for BAO-EVT. However, inherent limitations of CT/CTA for BAO strokes may lead to under-identification of patients who may derive significant benefit from EVT. Thus, for patients who appear to be poor candidates for basilar EVT due to limited or unfavorable CT/CTA findings, additional advanced imaging modalities may still be valuable as a second-line selection tool to potentially “rule in” their eligibility.

Our study has several limitations. Quantification of early ischemic signs (e.g. pc-ASPECTS or MPI) was not available in the STAR database, and it is unclear if patients had similar ischemic burdens between groups. Choice of imaging modality was based on local institutional policies, and the reasons for opting conventional versus advanced imaging selection was not recorded in the STAR database. It is possible that some patients who were selected for BAO-EVT may have undergone first-line conventional imaging that revealed concerning or uncertain findings. Thus, while our findings suggest that first-line conventional imaging may perform similarly well compared to advanced imaging modalities, we cannot rule out a clinical benefit of second-line advanced imaging for select BAO-EVT candidates. A large portion of BAO EVT patients in the STAR database did not have available information regarding the imaging modalities used to select each patient for treatment; thus, our study is vulnerable to ascertainment bias. Furthermore, while our study is the first to report a large series of patient outcomes of BAO EVT selected by conventional versus advanced imaging modalities, sample size is modest, and we may lack statistical power to detect more subtle differences. Finally, as a retrospective observational study with self-reported clinical and imaging outcomes without core lab adjudication, there may be uncaptured and hidden confounders that could not be accounted for.

## Conclusions

In patients with BAO, outcomes after EVT were similar in patients selected using conventional versus advanced imaging modalities. Thus, conventional imaging appears sufficient as a first-line tool for selecting basilar EVT patients in routine clinical practice. Future prospective studies are needed to investigate the role of advanced imaging modalities for BAO during acute triage, especially as a second-line modality for patients with equivocal or unfavorable conventional imaging findings.

## Supplementary Material

sj-tiff-1-eso_23969873251364973
