# Supplementary figures and images for: Conventional versus advanced imaging selection for endovascular treatment of basilar artery occlusion strokes
Source: Eur Stroke J. 2026 Jan 1;11(1):23969873251364973. doi: 10.1093/esj/23969873251364973 (PMC12866216; doi:10.1093/esj/23969873251364973)

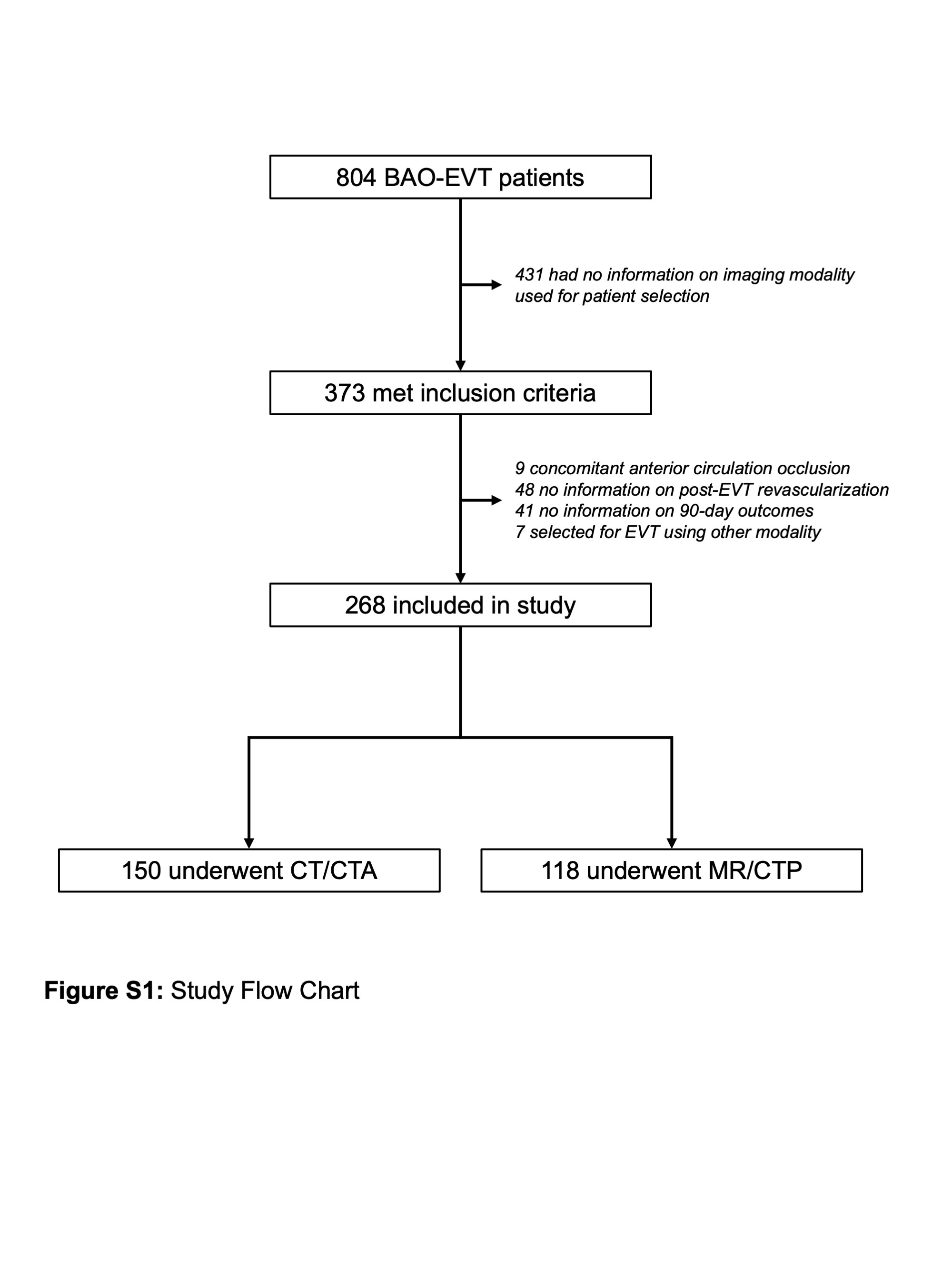

Supplement: sj-tiff-1-eso_23969873251364973 [file sj-tiff-1-eso_23969873251364973.jpeg]
